# Prevalence and pattern of acute and chronic multimorbidity across all body systems and age groups in primary health care

**DOI:** 10.1038/s41598-021-04256-x

**Published:** 2022-01-07

**Authors:** Michael Linden, Ulrike Linden, David Goretzko, Jochen Gensichen

**Affiliations:** 1 Research Group Psychosomatic Rehabilitation, Charité University Medicine Berlin, CBF, Hs.II, E01, Hindenburgdamm 30, 12200 Berlin, Germany; 2 Psychological Methods and Assessment, Ludwig-Maximilians-University Munich, Munich, Germany; 3 Institute of General Practice and Family Medicine, Ludwig-Maximilians-University Munich, Munich, Germany

**Keywords:** Medical research, Health care, Health services, Comorbidities

## Abstract

Multimorbidity is more than just the addition of individual illnesses, and its diagnosis and treatment poses special problems. General practitioners play an important role in looking after multimorbid patients. The aim of this study is to assess the prevalence and pattern of acute and chronic multimorbidity in primary care patients, regardless of body system and age group. A convenience sample of 2099 patients treated by 40 general practitioners was assessed using the Burvill scale. This measure of multimorbidity differentiates according to organ system and covers both acute and chronic illnesses. It also allows severity ratings to be assessed for both acute and chronic conditions, and thus patients’ actual need for general practice care. Patients reported an average of 3.5 (SD = 2.0) acute and/or chronically affected body systems. Overall, 12.7% of patients reported only one health problem, 83.0% at least two, 65.8% at least three, 46.1% at least four, and 29.7% five or more. The most frequent problems were musculoskeletal (62.5%) and psychological (56.6%). Some morbidities were interrelated, while others co-occurred despite being medically independent. In primary care, multimorbidity is the rule rather than the exception. Acute and chronic morbidity both contribute to the burden of illness. Body systems reflect treatment needs. Instead of specialist treatment for individual illnesses, an integrative treatment approach is needed. This is the specialty of general practitioners.

## Introduction

Primary care is responsible for the treatment of 40,000 to 60,000 different illnesses and health problems. In view of this high number and the prevalence rates of illnesses, many patients inevitably suffer from several disorders simultaneously. Such multimorbidity is a serious problem in modern and aging societies in which chronic illnesses are common^1–6^. Multiple health problems and their treatments can interfere with each other, leading to compounding negative effects^7^. Multimorbid patients are also high utilizers of medical care^8^.

The term “multimorbidity” means that several illnesses exist concurrently. These may or may not interact with each other. In the case of a “comorbidity”, the focus is on a specific illness, the treatment of which may be complicated by additional health problems. There is no final scientific consensus on how to measure or assess multimorbidity^8–15^. Physicians treating multimorbid patients face the problem of how many medical disciplines to take into account, who should conduct the assessments, how intensively patients should be examined, whether self- or expert assessments should be used, which laboratory tests should be performed, whether only the courses of illnesses (e.g. diabetes) or also their consequences (e.g. diabetic gangrene) should be taken into account, whether medically relevant problems (carcinoma) or also those with a subjective burden (warts) should be considered, whether health care utilizers or epidemiological samples should be described, whether acute or also chronic conditions should be included, and, last but not least, what thresholds (e.g. for initiating treatment for hypertension) should be used^16^. A comparatively simple method to describe treatment needs and to gain clinically meaningful information on the overall health status of a person is to ask which of his or her body systems have been affected. The Burvill scale^17^, which identifies acute and chronic illnesses and their severity, can be used for this purpose.

The aim of this study is to collect data on the role of multimorbidity in general practice for all body systems and age groups.

## Methods

### Setting

Practitioners in Berlin/Brandenburg, Germany, were contacted by phone and asked whether they would participate in the study. By this means, we obtained a convenience sample of 40 practitioners that, although not epidemiologically representative for all general practitioners in the area, were “prototypically representative”^18^, i.e. they represented practitioners that run well-functioning practices, are well established in their jobs, and are interested in the further development of their discipline. They had all undergone specialist training as general practitioners lasting at least 5 years before they were permitted to set up in private practice. The average age of the physicians was 52.3 years (SD = 7.5, range 38–71), and 59.0% were female. They had worked in their practices for an average of 12.6 (SD = 6.2) years and had treated an average of 1115 patients (range 350–2300) in the previous three months.

The German health care system does not foresee gatekeeping, and patients can directly contact any specialist in private practice, who will then be reimbursed by the patient’s health insurer^19^. For this reason, the present data are especially interesting, as they only refer to patients that intentionally sought health care from their general practitioner, and not to patients in the population as a whole.

Research assistants approached all patients in the waiting rooms of the participating general practitioners, and asked them to fill in several screening surveys. As a result of this methodology, patients that consult their practitioner on a regular basis—as opposed to those who only seek treatment when they have a particular problem—are over-represented^18,20^.

### Measure

Patients were given the Burvill survey. The survey lists ten body regions and gives short additional explanations for each of them^17^: (1) Cardiovascular system: e.g. hypertension, cardiac insufficiency, heart attack, arteriosclerosis, blood flow disorder or venous problems. (2) Endocrine system: e.g. thyroid dysfunction, diabetes, menopausal problems or liver diseases. (3) Respiratory system: e.g. allergic reactions, asthma, chronic bronchitis or sinusitis. (4) Genitourinary system: e.g. prostate problems, problems urinating, or insufficiency of the pelvic base. (5) Gastrointestinal tract: e.g. burping, gastric pain, intestinal illness, diarrhea, constipation or intestinal cancer. (6) Hematological/blood system: e.g. anemia, coagulopathy or special blood cells. (7) Ear and Eye: e.g. problems hearing, seeing, or conjunctivitis. (8) Musculoskeletal system: e.g. back pain, discus prolapse, pain in joints and muscles, bone fractures, rheumatism or arthrosis. (9) Nervous or neurological system: e.g. paralysis, stroke, brain tumor, multiple sclerosis or polyneuropathy. (10) Mental and psychological problems: e.g. depression, anxiety, petulance, fatigue or schizophrenia.

Patients were asked to rate acute and chronic problems for each named body system. Chronic problems were defined as having lasted longer than six months, and severity was rated on a four-point Likert scale from “(0) no illness “ to “(3) severe illness”. Patients were also asked for information on their age and gender. Such self-assessments represent what patients experience as health problems and what they are treated for. The information thus provides the clinical perspective of care, and does not reflect what the results of a comprehensive interdisciplinary medical examination would have been.

### Analysis

First, the proportion of participants reporting an illness was separately calculated for each body region and according to whether the problem was acute or chronic. To shed light on different multimorbidity patterns, co-occurrences were then analyzed. Besides this descriptive approach, we also used exploratory graph analysis (EGA) to further explore the co-occurrences of medical issues in different body regions^21^. EGA is based on a network model (a Gaussian graphical model) and models covariance and correlations between variables. In our case, patients’ ratings for each body region (acute and chronic) are treated as the nodes, and edges in the network represent partial correlations between them. Using the *R* package EGAnet^22^, we conducted EGA, whereby we selected the regularization parameter based on the EBIC (model arguments: gamma = 0.5, lambda.min.ratio = 0.01, refit = TRUE) and conducted bootstrapping (500 iterations) to stabilize the solution.

All physicians and patients gave their informed consent in writing. There were no subjects with a guardian or below the age of 18. All methods were carried out in accordance with relevant guidelines and regulations. There were no experimental protocols.

The study protocol was reviewed for the fulfillment of ethical, data security, and legal requirements by the internal scientific review board of the Federal German Pension Agency and was also revised and approved by the ethical committee of the Charité University Medicine Berlin (EA4/097/09).

## Results

A total of 2987 patients were approached in the waiting rooms, of whom 888 patients declined to participate in the study, and the remaining 2099 were included. The participants were between 18 and 89 years old (MW = 46.4, SD = 16.1), and 62.6% of them were female. The mean age of the females was 46.7 (SD = 16.0) and the mean age of the males 45.8 (SD = 16.3) years.

**Figure 1:**
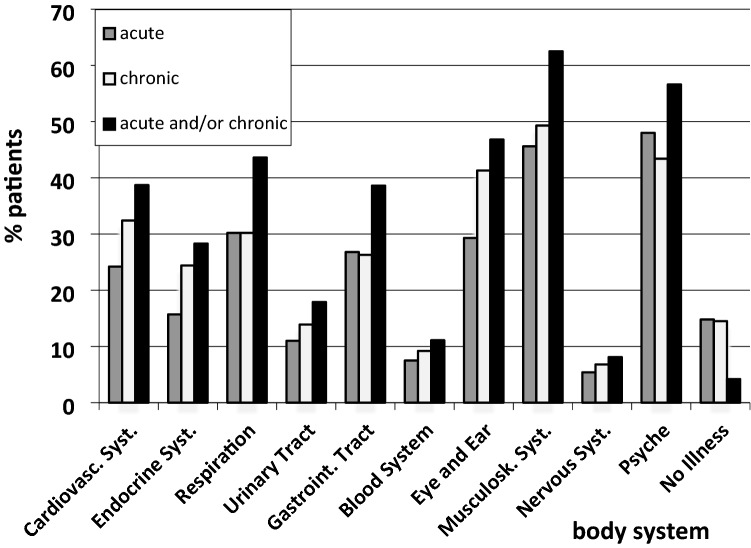
Percentage of patients awaiting a GP consultation according to acute and chronic disease and impaired body system.

Figure 1 shows the percentage of patients with acute and chronic illnesses per body system. Overall, 4.2% of patients reported no illness, reflecting that GPs are not only contacted because of existing illnesses but because health certificates etc. are required. The most frequent problems were musculoskeletal (62.5%), psychological (56.6%), or with eyes/ears (46.8%). The least frequent were neurological (8.1%), blood (11.1%), and urogenital problems (17.9%).

**Figure 2:**
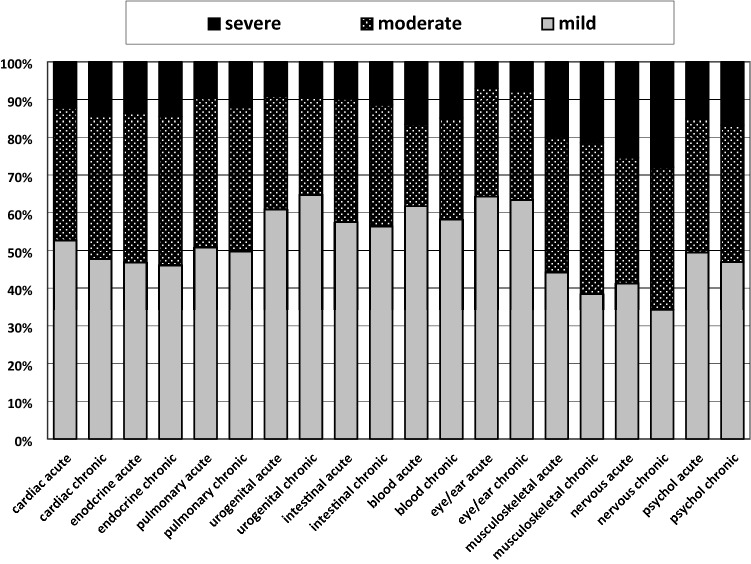
Severity ratings for acute and chronic problems by body system.

Figure 2 shows the severity ratings for acute and chronic problems per body system. About half the problems were rated mild, and ten to twenty percent severe. The most severe ratings were given to musculoskeletal, neurological and psychological problems. There is no pronounced difference between the prevalence of acute and chronic problems.

**Figure 3:**
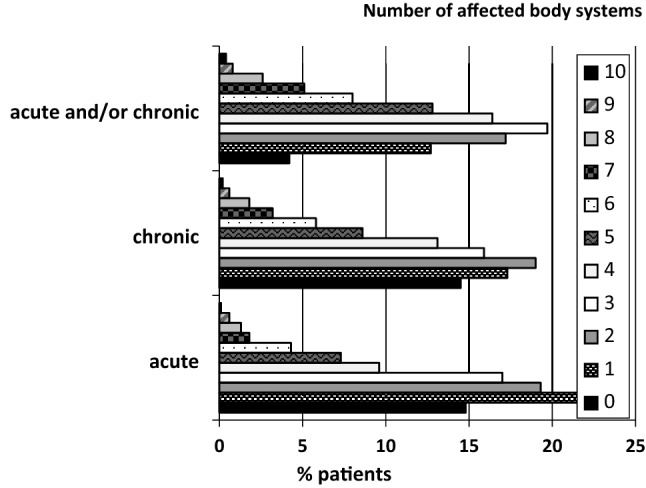
Percentage of patients with acute and chronic diseases according to the number of affected body systems (1–10).

On average, patients reported acute health problems in 2.4 (SD = 2.0) and chronic health problems in 2.8 (SD = 2.1) body systems, as well as an overall 3.5 (SD = 2.0) acutely/chronically affected body systems out of the 10 presented in the instrument. Of the patients, 12.7% had only one health problem, 83.0% at least two, 65.8% at least three, 46.1% at least four, 29.7% at least five, and 16.9% six or more (Fig. 3).

There is a difference in multimorbidity prevalence between age groups. Patients under the age of 30 had on average 1.86 (SD 1.54) acute, 1.75 (SD = 1.57) chronic and 2.59 (SD = 1.67) acute/chronic problems, with 30.3% reporting none, or only one health problem. Patients between 31 and 60 years of age had an average of 2.42 (SD = 1.92) acute, 2.87 (SD = 2.12) chronic and 3.64 (SD = 2.04) acute/chronic problems, with 15.1% having none, or only one problem. Patients aged 60 or older had an average of 5.50 (SD = 2.11) acute, 3.04 (SD = 2.23) chronic and 4.12 (SD = 2.03) acute/chronic problems, with 8.5% saying they had only one problem. Nevertheless, there is even a considerable number of younger persons with more than 3 simultaneously affected body systems (< = 30 years: 27.5%, 31–60 years: 48.7%, 60 + years: 57.7%).

Females had on average 2.54 (SD = 2.00) acute, 2.94 (SD = 2.14) chronic, and 3.71 (SD = 2.04) acute/chronic problems, with 14.5% reporting none, or only one problem, and 51.2% more than three. Males reported an average of 2.25 (SD = 1.88) acute, 2.49 (SD = 2.01) chronic, and 3.20 (SD = 1.99) acute/chronic problems, with 20.9% reporting none, or only one problem, and 37.8% more than three. Taken as a whole, females reported more health problems than males in terms of overall morbidity (acute/chronic: Chi^2^ 26.21, *p* < 0.001, chronic: Chi^2^ 39.71, *p* < 0.001, acute: Chi^2^ 12.99, *p* = 0.011).

Table 1 gives an overview on the pattern of comorbidity. For example, 74.0% of patients with an acute cardiac problem also had a chronic cardiac problem, while 34.1% also had an acute and 78.7% a chronic endocrine problem, 41.1% an acute and 38.9% a chronic pulmonary problem etc. Comorbidity rates above 70% were found for acute cardiac and chronic cardiac problems (74.0%), and for chronic endocrine problems (78.7%). Acute endocrine problems were comorbid with chronic endocrine problems (74.8%), chronic urogenital problems with chronic musculoskeletal problems (76.4%), and acute blood problems with chronic blood (74.5%) and acute musculoskeletal problems (70.7%). Acute musculoskeletal problems were comorbid with chronic musculoskeletal problems (71.0%). Acute neurological problems were comorbid with acute musculoskeletal problems (73.7%), with chronic neurological problems (77.2%), and with acute (70.2%) and chronic psychological problems (71.9%). Chronic neurological problems were comorbid with chronic musculoskeletal problems (75.5%) and chronic psychological problems (78.3%). Acute psychological problems were comorbid with chronic psychological problems (72.3%), and chronic psychological problems with acute psychological problems (80.0%). The smallest comorbidity rates were found for the comorbidity of pulmonary problems with neurobiological problems (5.9% to 8.8%) and of psychological problems with neurological problems (7.9% to 12.3%).

**Table 1:** Percentage of patients per disease (row) that have a comorbidity from another body system (column) (e.g. from 507 patients with acute cardiac problems, 74.0% also have chronic cardiac problems and 34.1% acute endocrine problems).

BS N_n_	Car_a 507	Car_c 681	End_a 329	End_c 512	Pul_a 634	Pul_c 639	Uro_a 230	Uro_c 292	Int_a 563	Int_c 553	Blo_a 157	Blo_c 194
Car_a		55.1	52.6	38.3	32.8	30.8	52.2	39.0	34.1	30.0	49.0	40.2
Car_c	74.0		52.0	53.5	33.9	43.7	50.0	59.2	34.5	45.8	45.2	52.6
End_a	34.1	25.1		48.0	21.3	20.7	34.8	26.0	24.0	23.1	37.6	29.4
End_c	78.7	40.2	74.8		25.7	33.2	32.6	41.8	26.5	34.0	40.1	41.2
Pul_a	41.0	31.6	41.0	31.8		55.9	40.4	33.6	37.8	34.5	43.9	32.5
Pul_c	38.9	41.0	40.1	41.4	56.3		39.1	41.4	33.7	44.1	34.4	40.7
Uro_a	23.7	16.9	24.3	14.6	14.7	14.1		50.5	21.0	16.6	23.6	19.1
Uro_c	22.5	25.4	23.1	23.8	15.5	18.9	63.5		19.7	28.2	24.2	26.3
Int_a	37.9	28.5	41.0	29.1	33.6	29.7	51.3	38.0		55.2	46.5	38.1
Int_c	32.7	37.2	38.9	36.7	30.1	38.2	40.0	53.4	54.2		39.5	44.8
Blo_a	15.2	10.4	17.9	12.3	10.9	8.5	16.1	13.0	13.0	11.2		60.3
Blo_c	15.4	15.0	17.3	15.6	9.9	12.4	16.1	17.5	13.1	15.7	74.5	
Eye_a	51.7	39.4	53.2	36.3	35.5	32.4	53.5	41.1	43.7	37.3	57.3	43.8
Eye_c	53.6	56.8	59.3	55.3	32.3	48.4	57.4	68.5	49.6	53.7	56.7	59.3
Mus_a	62.5	51.8	65.3	51.0	57.1	50.1	68.7	55.8	60.9	53.0	70.7	56.2
Mus_c	63.7	66.7	66.9	65.4	55.2	63.1	61.7	76.4	56.0	67.8	67.5	68.0
Neu_a	10.5	8.1	12.2	8.0	6.8	5.9	11.7	9.9	10.1	9.6	17.2	12.9
Neu_c	10.8	12.2	12.8	11.5	6.5	8.8	14.8	14.4	9.9	12.7	15.9	17.0
Psy_a	56.4	50.1	65.7	56.3	57.6	53.5	61.3	56.8	64.5	59.3	65.6	58.2
Psy_c	51.7	51.8	58.4	56.1	47.6	56.0	51.7	59.6	56.7	65.6	59.2	63.9

BS N_n_	Eye_a 614	Eye_c 867	Mus_a 958	Mus_c 1034	Neu_a 114	Neu_c 143	Psy_a 1007	Psy_c 910				
Car_a	42.7	31.4	33.1	31.2	46.5	38.5	28.4	28.8				
Car_c	43.6	44.6	36.8	43.9	48.2	53.1	33.9	38.8				
End_a	28.5	22.5	22.4	21.3	35.1	29.4	21.4	21.1				
End_c	30.3	32.6	27.2	32.4	36.0	41.3	28.6	31.5				
Pul_a	36.6	30.9	37.8	33.8	37.7	28.7	36.2	33.2				
Pul_c	33.7	35.6	33.4	39.0	33.3	39.2	34.0	39.3				
Uro_a	20.0	15.2	16.5	13.7	23.7	23.8	14.0	13.1				
Uro_c	19.5	23.1	17.0	21.6	25.4	29.4	16.5	19.1				
Int_a	40.1	32.2	35.8	30.5	50.0	39.2	36.0	35.1				
Int_c	33.6	34.3	30.6	36.3	46.5	49.0	32.6	39.9				
Blo_a	14.7	10.3	11.6	10.3	23.7	17.5	10.2	10.2				
Blo_c	13.8	13.3	11.4	12.8	21.9	23.1	11.2	13.6				
Eye_a		57.6	43.3	37.1	45.6	40.6	35.6	33.5				
Eye_c	81.3		47.0	53.2	52.6	56.6	42.8	49.3				
Mus_a	67.6	51.9		65.8	73.7	58.7	56.5	53.5				
Mus_c	62.5	63.4	71.0		69.3	75.5	56.9	65.6				
Neu_a	8.5	6.9	8.8	7.6		61.5	7.9	9.0				
Neu_c	9.4	9.3	8.8	10.4	77.2		9.7	12.3				
Psy_a	58.3	49.7	59.4	55.4	70.2	68.5		80.0				
Psy_c	49.7	51.8	50.8	57.7	71.9	78.3	72.3					

BS, body system; a, acute; c, chronic; car, cardiac; end, endocrine; pul, pulmonary; uro, urogenital; int, gastrointestinal; blo, blood; eye, eye/ear; mus, musculoskeletal; neu, neurological; psy, mental/psychological; N, number of patients with disorder.

**Figure 4:**
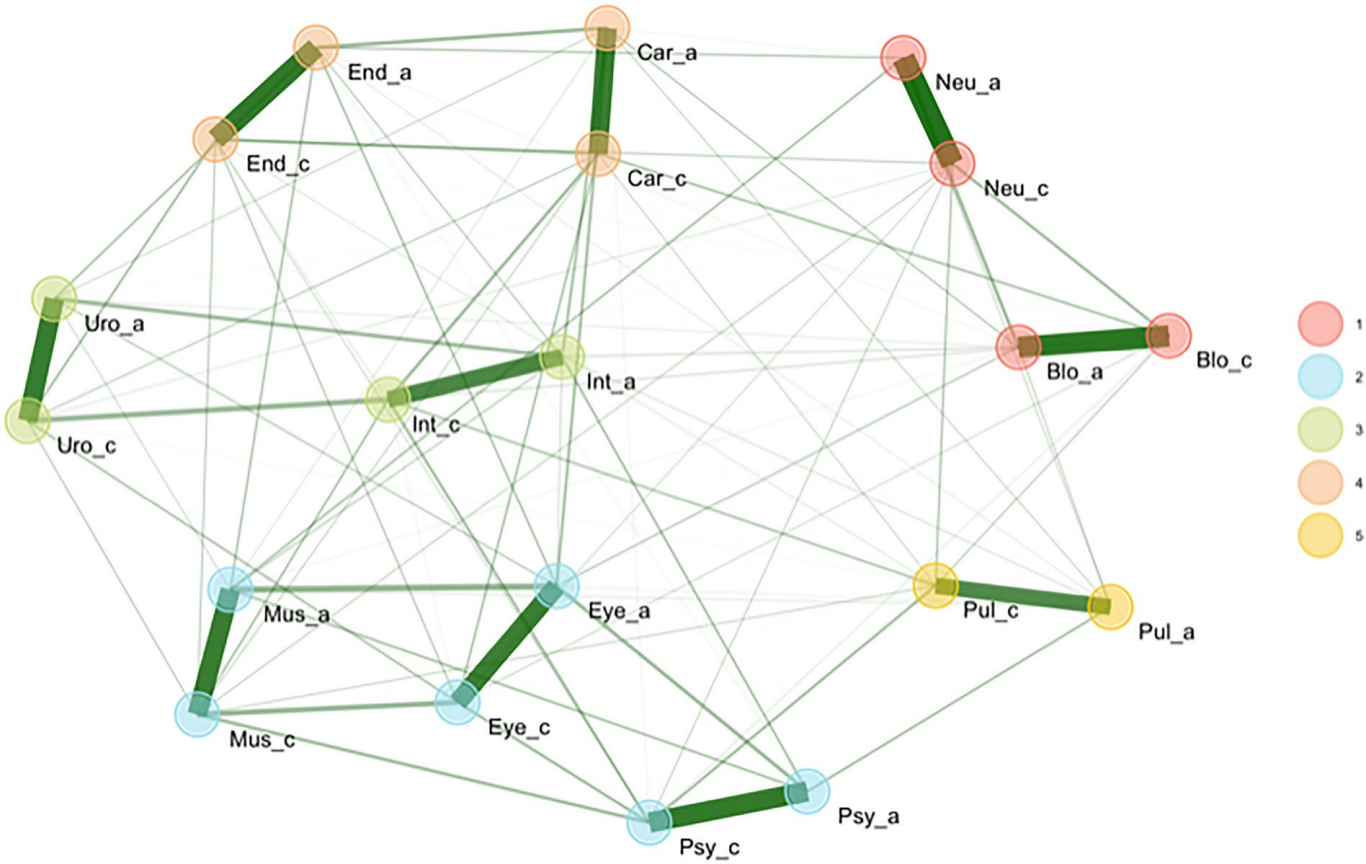
Co-occurrence and clusters (1–5) of acute and chronic health problems in the ten body regions under investigation (_a = acute, _c = chronic, Car = cardiac, End = endocrine, Pul = pulmonary, Uro = urogenital, Int = gastrointestinal, Blo = blood, Eye = eye/ear, Mus = musculoskeletal, Neu = neurological, Psy = mental/psychological).

EGA indicates that the most strongly related comorbidities were between acute and chronic health issues in the same body region (thickness of lines in Fig. 4). Furthermore, analysis suggests that the co-occurrence of health issues in specific body regions are strongest in five clusters. Strong associations exist between the cardiovascular and endocrine systems (Fig. 4.: cluster #4), urogenital and gastrointestinal systems (cluster #3), and musculoskeletal, hearing/seeing and psychological systems (cluster #2). The respiratory system formed its own cluster (cluster #5) and did not show particularly strong links to other body regions.

## Discussion

As discussed above, conceptual and practical problems are involved in defining and measuring multimorbidity. We used the Burvill scale^17^, a measure of multimorbidity that (a) is organized according to organ system and is a clinically valid framework, (b) includes both acute and chronic illnesses, in contrast to most of the literature, which focuses only on chronic illnesses and thus fails to represent the actual care needs of people consulting a general practitioner, (c) rates severity for both acute and chronic conditions, and (d) represents the actual care needs of patients consulting a general practitioner and reflects the reality of this medical discipline.

The results of our study confirm those of other studies showing the high prevalence of multimorbidity in general practice patients. On average, patients have health problems in 3.5 body systems, while only 12.7% have only one problem, and 83.0% complain about issues affecting at least two body systems. Almost every second patient has four or more problems at the same time. Patients with only one illness are the exception. Our rates are higher than those reported in other studies (34% to 61% of multimorbid patients), official statistics and reimbursement systems^2,8,13^. However, our rates include mild health problems in addition to acute and chronic illnesses, which is important, as combinations of mild and chronic, and mild and acute disorders, can create problems of their own.

As is to be expected, the prevalence of multimorbidity is higher in the elderly and in female patients^2,16^, but young patients with multimorbidity also exist.

Musculoskeletal and psychological disorders are most often reported. Both frequently occur in the general population and therefore in general practice. Family physicians therefore need a thorough education in these two areas. The awareness of some illnesses with mild symptoms and little impairment (e. g. hypertension) is often low, and may therefore be underrepresented in this study. This may be the reason that only 32.4% of GP patients reported having chronic cardiovascular disease.

The comorbidity patterns shown in Table 1 reveal that some patients have health problems in as many as ten body systems (0.4%). In ten body systems, it is possible to have one of 1,233,311 different disorder combinations. The interrelations shown in Table 1 and similarly in the EG, which considers only “unique” bivariate association structures while controlling for other covariates (body regions), show highly plausible patterns. Persons who have a chronic illness in a specific body system are more likely to have acute conditions in the same system than those with no chronic conditions. Similarly, the relations between different body systems are well understood, and it is well known that musculoskeletal and psychological complaints often occur together, as do endocrinological and cardiac problems. The reported clustering of body regions may be helpful in the future investigation of common comorbidities.

## Conclusion

Multimorbidity is the rule rather than the exception in primary care patients, and the problem is becoming more serious as society ages^1,2,16^. The predominant problems are musculoskeletal and psychological, and they are associated with much subjective suffering for the patient. General practitioners must therefore master the full range of medical specializations. As the possibilities are so numerous, controlled clinical trials and treatment guidelines will never be available for all possible combinations of multimorbidity, and most medical guidelines and educational programs focus on individual illnesses, so their validity is limited in general practice^9,15,23^. As multiple health problems require different medical approaches than individual illnesses, general practice is a medical specialty in its own right. The high rate of occurrence of chronic conditions should also be taken into account in the organization of care, and supports the view that general practice is perhaps the most important discipline in overall healthcare^24,25^.

## Limitations

The sample of general practitioners is not representative of all general practitioners in all countries, and results may vary depending on the health care system and population under investigation. Our results are based on the self-reports of patients. If specialists had carried out a thorough medical assessment of the patients, the results may have been different.
